# The retrieval of unerupted teeth in pedodontics: two case reports

**DOI:** 10.1186/1752-1947-8-334

**Published:** 2014-10-09

**Authors:** Simona Tecco, Mariano Lacarbonara, Maria Teresa Dinoi, Gianni Gallusi, Enrico Marchetti, Stefano Mummolo, Vincenzo Campanella, Giuseppe Marzo

**Affiliations:** 1 University Vita-Salute San Raffaele, Dental Clinic, via Olgettina, 58 20132 Milano, Italy; 2 Department of Life, Health and Environmental Sciences, School of Dentistry, University of L’Aquila, L’Aquila, Italy; 3 Department of Clinical Sciences and Translational Medicine, School of Dentistry, University Tor Vergata, Rome, Italy

**Keywords:** Impacted lower canine, Impacted lower first molar, Radiological diagnosis

## Abstract

**Introduction:**

The retrieval of unerupted teeth in pedodontics is always significant to preserve the trophism of adjacent tissues, establish the correct space, provide adequate function and maintain good esthetics for the patient. The treatment plan is based on radiographic examinations and measurements, and on an accurate clinical evaluation; it aims to achieve the best treatment possible depending on the complexity of the specific case.

In the most difficult clinical cases it is very important to have an early diagnosis, which is essential to plan the treatment and achieve success. In these cases, the pediatrician is in a strategic position to give an early diagnosis through a child’s medical history and by counting the child’s teeth.

**Case presentation:**

This article presents two different difficult clinical cases of impacted teeth diagnosed during pediatric age, with a radiological analysis, and successfully treated with orthodontic devices designed for these specific cases. Clinical case 1 describes a 13-year-old Italian girl; clinical case 2 describes a 9-year-old Italian girl. The use of these devices achieved the desired treatment goals. The problems associated with impacted teeth and the biomechanical interventions used for these patients are discussed.

**Conclusions:**

An early and careful diagnosis followed by an accurate treatment plan for the individual cases can lead to retrieval of the impacted teeth without affecting other anatomic structures and adjacent teeth. In these cases, the pediatrician is in a strategic position to give an early diagnosis through a child’s medical history and by counting the child’s teeth.

## Introduction

The eruption of permanent teeth in the dental arch is regulated by a significant genetic control [1] and this guides the correct formation of tooth buds and their eruption in the dental arch in their right positions.

Certain anatomical conditions or previous traumas or affections of the corresponding deciduous tooth, may lead to eruption anomalies in terms of time or position, or in some cases can arrest completely the physiological eruption of the permanent tooth (dental inclusion).

The pediatrician is certainly the first physician to visit young patients and, as such, may be able to intercept all oral diseases. The pediatrician must provide general information to prevent the onset of caries, through proper nutrition and proper use of fluoride. The pediatrician may ask parents to make a dental visit and then implement all the measures of prevention as ambulatory care (for example, the sealing of the first permanent molars) [2].

It is important that pediatricians know the importance of normal oral growth and development. Often the parents of a young patient ask their pediatrician to assess which is the right time to refer their child to a dental visit, or even orthodontics. This is the reason why it is better that the pediatrician is aware of complications that arise from the inclusion of permanent teeth, which can be prevented and cured when the patient is a child. In the most difficult clinical cases of impacted teeth it is very important to have an early diagnosis, which is essential to plan the treatment and achieve success. The pediatrician is in a strategic position to give an early diagnosis through a child’s medical history and by counting the child’s teeth.

A tooth is referred to as “retained” when it has not erupted in the dental arch within its physiological time but still shows radiographic evidence of eruptive capacity and has no anatomic obstruction on its eruptive path [3,4]. A tooth is referred to as “impacted” if it is completely or partially unerupted many years after normal eruption time or if it is positioned against another tooth, bone or soft tissue, so that its further eruption is unlikely [3,4]. The position of these teeth can often show a very marked ectopy [3,4].

Some studies demonstrated that the incidence of dental impactions ranges from 5.6% to 18.8% with a higher frequency among women [5].

Teeth that most frequently face impactions are the lower and upper third molars (20 to 30%). Third molars, in order of frequency, are followed by upper canines (85% with palatal dislocation) which first face retention and then impaction. Upper canines are followed by lower second premolars (0.3%) that usually face the impaction because of the premature eruption of the first molar and the first premolar [6,7]. Upper central incisors (0.1%) represent the rarest case of impacted teeth [7,8].

To formulate a prognosis and a treatment plan it is necessary to consider the different aspects of impactions.

Depending on the grade of impaction there can be a distinction between complete or partial impaction. Partial impaction occurs when at least a portion of the crown is visible in the dental arch. Complete impaction occurs when the crown is not visible; it may be: endosteal, where the tooth is impacted completely within the bone; osteomucosal, where the tooth is completely covered by mucosa and partially by bone and mucosal, where the tooth is covered only by mucosa [9].

Depending on the number of impacted teeth there is a distinction between single impaction and multiple impactions [9].

Based on the duration the impaction of a tooth can be defined as temporarily impacted or permanently impacted [10]. Temporary impaction relates to a retained tooth caused by an obstacle (odontoma, cyst or supernumerary) that, as the obstruction is removed, erupts spontaneously in the dental arch [10]. By contrast, the impaction is permanent when surgical-orthodontic treatment is necessary to obtain eruption although the obstacle has been removed.

Finally, impaction can be primary or secondary depending on its cause [11]. Primary impaction is due to dental intrinsic factors (such as anatomy, inclination), whereas secondary impaction is caused by external factors such as cystic pathologies, supernumerary or neoformations [11].

The etiopathogenesis of impactions is very broad and causes are divided into general, local and structural.

• General causes can be: hereditary, hypofunctional endocrine disorders (hypothyroidism, pituitary cretinism), hyperfunctional disorders (hyperthyroidism), dysmetabolic conditions (hypovitaminosis and rachitis) and infectious diseases (congenital syphilis, rubella, scarlet fever) [12].

• Local causes can be related to the deciduous tooth (persistence, ankylosis, premature loss, chronic periapical inflammation) or associated with the permanent tooth (radicular ankylosis, coronal or radicular morphological alterations, position anomalies, eruption pattern anomalies) [13].

• Structural causes are maxillary hypoplasia, severe hyperdivergence, skeletal open bite [13,14] and congenital disorders of the maxillofacial apparatus such as labiopalatoschisis, cleidocranial dysostosis, cranial stenosis and Down’s syndrome [4,15,16].

The suspect of impaction or retention of one or more teeth can be derived from an accurate clinical examination, and family and personal medical history.

Inspection and palpation by a dentist may complete the clinical examination. The final diagnosis and prognosis can be done by an orthodontist with the support of an X-ray examination that shows the presence and the position of one or more unerupted teeth [4,17,18].

Useful radiographs in the diagnosis of impaction are panoramic, occlusal or periapical X-ray, or for high accuracy or surgical planning conventional computed tomography (CT) scans or cone beam CT scans. The orthopanoramic radiograph provides diagnostic certainty of the impacted tooth, giving an idea of its position and inclination and its relations with adjacent anatomical structures but it lacks the third dimension in understanding the precise position of the impacted tooth. In adjunct to the panoramic examination, an occlusal projection allows a more accurate determination of the position of the impacted tooth. Currently, the most precise X-ray examinations to reveal the position of the impacted tooth and of the other nearby anatomical structures, are conventional CT scans and low-radiation cone beam CT scans [19].

There are many different types of treatment options: classic orthodontic treatment; combined surgical-orthodontic treatment; preservative-surgical treatment; and radical surgical treatment [13]. When the tooth is retained for a matter of space, only a classic orthodontic interceptive treatment is performed. When the tooth is impacted and shows abnormal inclination and position, or has a particular coronal-radicular morphology a combined surgical-orthodontic procedure is required. When tooth eruption is blocked by a pathological condition (such as cysts, odontomas, and so on), its eruption in the dental arch depends on the removal of the obstacle; this is the preservative-surgical procedure (removal of the obstacle). Only in extreme situations, and in the presence of severe anatomical or positional anomalies, a radical surgical treatment may be chosen (removal of the impacted tooth) with the agreement of the patient.

The interceptive retrieval of an impacted tooth gains in importance particularly during the developmental age to guarantee the trophism of adjacent tissues, to maintain space, for esthetic and functional reasons. Even in the case that the retrieved tooth does not guarantee a long-term result, the procedure is advisable within limits. In that case the retrieved tooth with no long-term prognosis will perform its function until the patient reaches the age for prosthetic substitution of the tooth.

To prevent impactions different types of dental extraction can be performed such as, serial extractions, extractions of unexfoliated or ankylosed deciduous teeth and extraction of supernumeraries.

Complications that might occur after dental impactions can be distinguished between mechanical (resorption of the adjacent tooth roots, decubitus), nervous, infective (lower third molar pericoronitis, periodontal diseases, root resorptions of the adjacent tooth) [10,20] and dysplastic (follicular cysts, keratocysts, ameloblastoma) [4,9,11,21].

Thus, the choice of the optimal treatment strategy depends on a correct diagnosis and the pedodontic-orthodontic approach.

As stated above, there are prevention methods against impactions that, however, are to be promptly carried out.

A radiographic screening at an early age is able to intercept dental retention allowing prompt treatment.

The more an impacted tooth is situated far from its correct position or with a seriously tilted axis the gentler and more time consuming will be the orthodontic movement to reposition it. Maximum care will be necessary to avoid damage to adjacent teeth. Connecting the traction device directly to the orthodontic arch will produce an excessive force on the teeth adjacent to the impacted one leading to unwilled traumas or movements [4]. In these cases the use of auxiliary devices working with maximum anchorage to unload the teeth from traction counterforce is indicated [4].

Assessing the position and path of eruption of an unerupted tooth from a true lateral skull, orthopantomograph or a standard occlusal radiograph is considered clinically important for developing a comprehensive treatment plan. Several studies have recommended many radiological parameters of practicability to bring about speedy treatment and its effective resolution. For the lower impacted canine, a problem exists with the transmigration of the impacted tooth. Howard observed that those unerupted canines that lie between 25° and 30° in the midsagittal plane do not migrate across the mandibular midline. Those canines that lie between 30° and 95° tend to cross the midline. An overlap appears to exist between 30° and 50°. When the angle exceeds 50°, crossing the midline becomes a rule [22]. For the transmigrated canine, extraction or transplantation can be proposed.

It was stated that if the apex of the lower canine is seen to have migrated past the apex of the adjacent lateral incisor, it might be mechanically impossible to bring it into place [23].

Among radiological parameters, it was also suggested that it may be impossible to bring the impacted lower canine to its correct position in the presence of an overly mesially angulated unerupted canine that has begun to migrate labially across the incisors [24].

For the impacted first permanent molar, there is no clear standard solution for how to treat retained or impacted first molars, as treatment depends on several local factors such as the angulations/inclination of the impacted/retained tooth [25].

Although these previous articles mentioned and discussed various principles for treating practicable impacted teeth, the treatment of impacted teeth out of recommended radiological parameters of practicability has rarely been reported.

In this report, two clinical cases are described in which impacted teeth out of recommended radiological parameters of practicability were treated orthodontically with new purposely conceived orthodontic devices, which achieved the desired treatment goals.

## Case presentation

### Clinical case 1

A 13-year-old Italian girl was referred by her pediatrician because of a retained deciduous canine in her right mandible. During an earlier visit to the pediatrician, the doctor, considering the age of the patient, asked her about the exchange of deciduous teeth, and she reported that the tooth had not yet changed. She was not alarmed, neither was her mother, but the pediatrician insisted that the tooth would probably have already dropped. The pediatrician therefore encouraged her to contact her dentist.

The girl was in good health, and her dental and medical history was unremarkable with only the usual childhood maladies.

**Figure 1:**
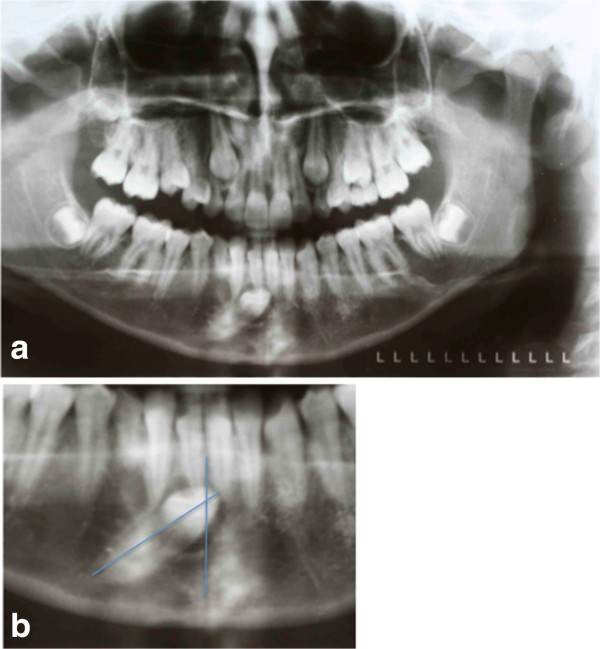
**Pretreatment records. (a)** Pretreatment panoramic radiograph. **(b)** The unerupted canine is going to migrate across the mandibular midline, and its crown tip is near the apex of the lower right first incisor root.

**Figure 2:**
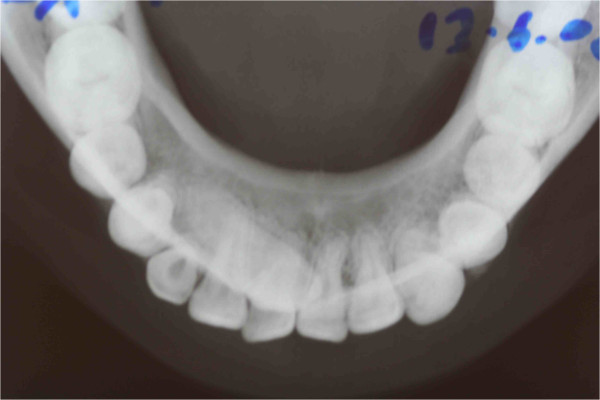
The occlusal radiograph confirms that the crown of the impacted canine is vestibular.

**Figure 3:**
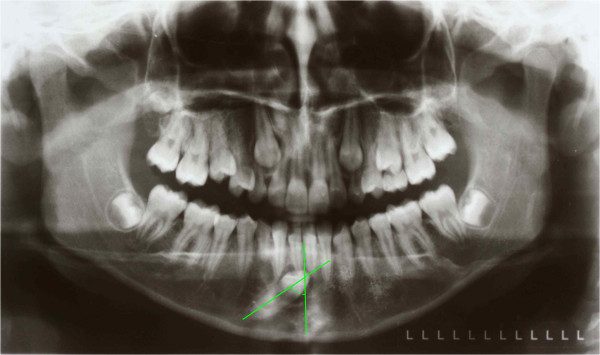
The canine angulation to the midline is 55°.

An extraoral clinical examination disclosed a symmetrical face with balanced vertical thirds.She shows a dental-skeletal class I with normal mandibular divergence, with no bad habits, and her cephalometric values are all normal; even her lower and upper incisors are normal-inclined. Her profile is standard for Italian people. An intraoral examination revealed that her dental midlines were concordant with each other and with her face, and no mandibular shift was detected on closure. Except for some lower incisor crowding the overall occlusion was fair with acceptable overjet and overbite. Her clinical periodontal parameters were normal. A radiographic examination revealed that the mandibular right canine was in an oblique position with its crown tip near the apex of the lower right first incisor root (Figure 1). An occlusal radiograph confirmed that the crown of the impacted canine was vestibular (Figure 2). The canine angulation to the midline was 55° (Figure 3). This value suggested a very difficult problem, which might not be orthodontically treatable. After careful evaluation of this case, in view of the age of the patient, the clinical decision was to treat this impacted tooth orthodontically. Full mouth orthodontic treatment was suggested.

**Figure 4:**
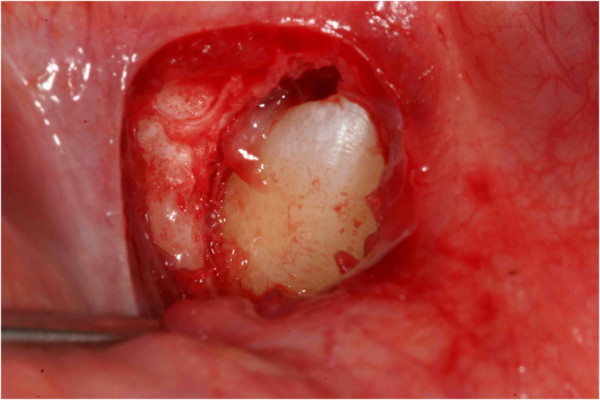
Surgical outbreak; a vestibular repositioned, full thickness mucoperiosteal flap is elevated, and the crown of the canine is exposed.

**Figure 5:**
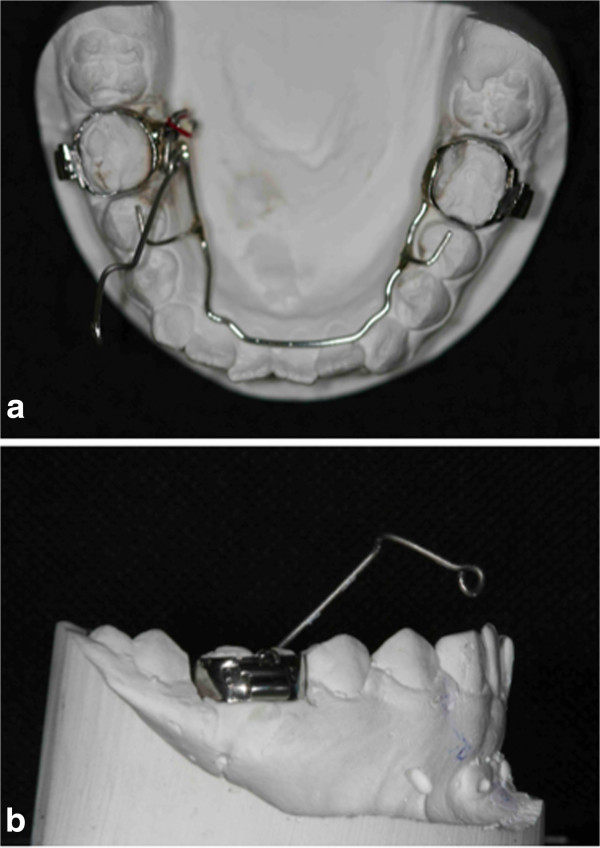
**The orthodontic devices: the Fishing-rod. (a)** Fishing-rod in occlusal view. **(b)** Fishing-rod in lateral view. The lever arm of the device allows for a push in the occlusal-distal direction of the crown of the impacted tooth.

**Figure 6:**
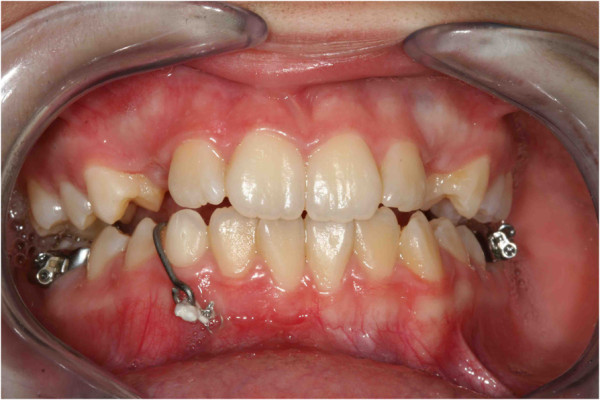
Intraoral photographs with fishing-rod; the appliance is used to tie up and drive into the canine’s eruption.

**Figure 7:**
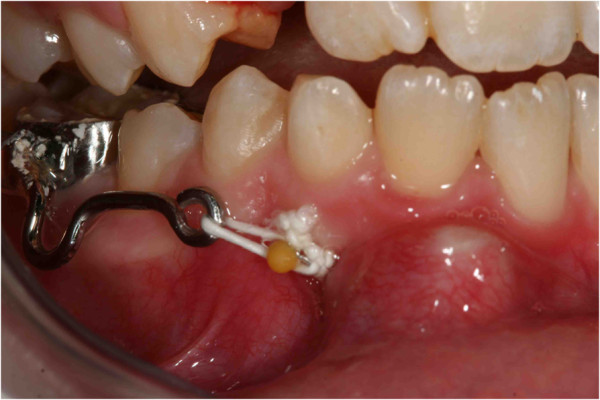
After 5 months, the cusp of the canine was visible in the mouth.

**Figure 8:**
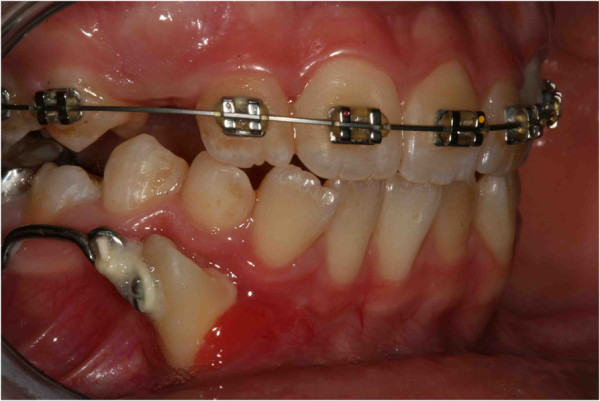
After 8 months; pre-informed brackets and straight archwires are used.

**Figure 9:**
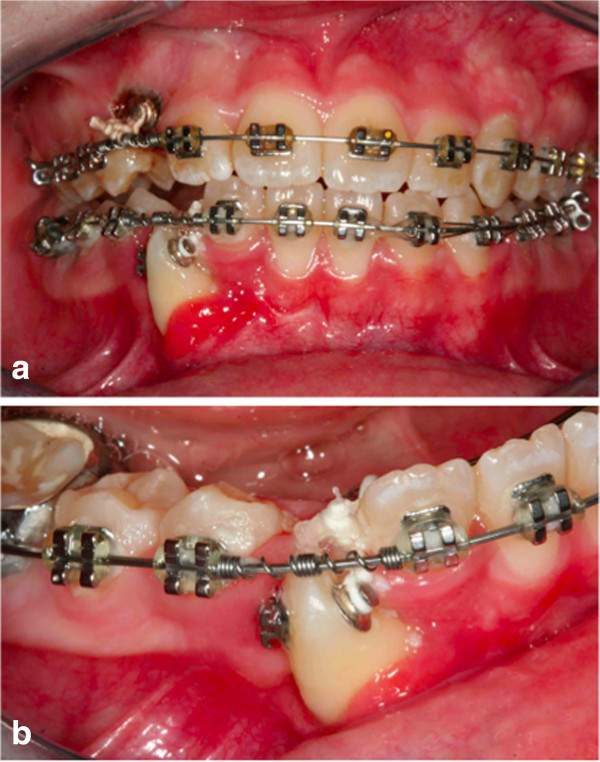
**After 12 months.** When the canine was present in the oral cavity **(a)**, a bracket was bonded to it and linked directly to the arch by an elastic ligation. To keep the space in the arch for the canine, since it was not aligned, an open coil spring **(b)** was used.

**Figure 10:**
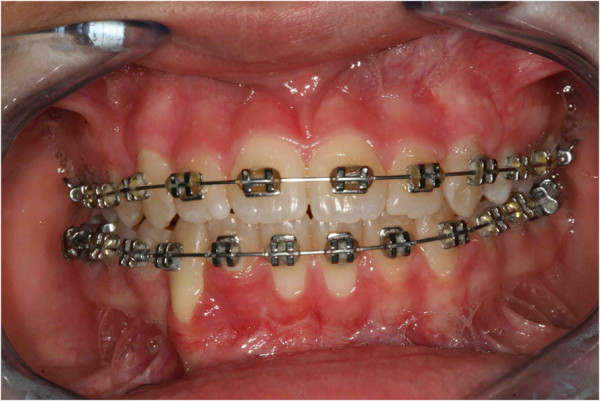
After 18 months the canine is well positioned in the arch.

**Figure 11:**
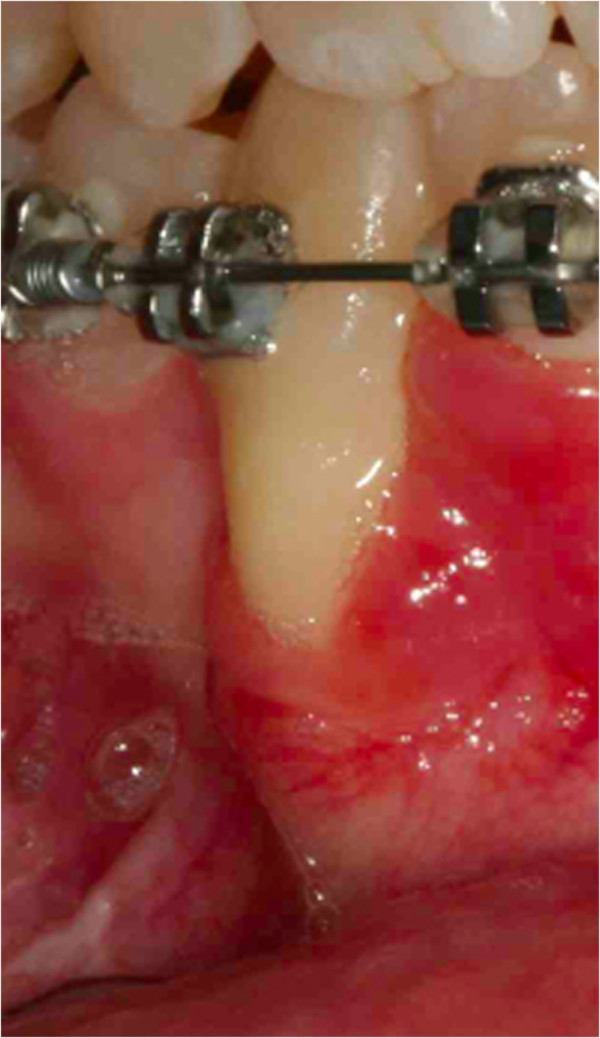
After 18 months the patient was advised that she needed an attached gingiva graft on the restored tooth to improve esthetics and the periodontal health compromised by the treatment.

It was our goal to treat this case with a non-extraction orthodontic approach using upper and lower jaw appliances, while doing our best to correct the impacted tooth, to maintain the profile and reaching as good a final occlusion as possible. The objectives of orthodontic treatment for this patient were to bring the impacted mandibular right canine into her dental arch, level and align the arches, maintain the normal overjet and overbite, and achieve a bilateral Class I canine and molar occlusion. First, the oral surgeon had to eliminate the retained mandibular deciduous canine. At the same time, a vestibular repositioned, full thickness mucoperiosteal flap was elevated, and the crown of the canine was exposed (Figure 4). In the same session a fishing-rod (it is a lingual arch of bands positioned on the first permanent molar with the addition of an arm in titanium-molybdenum alloy wire with a trend from the lingual to the buccal side; it is used for traction on canines) was cemented (Figure 5); this appliance is fixed, and previously prepared by the technician; the appliance was used to tie up and drive into the canine’s eruption (Figure 6). After 5 to 8 months, the cusp of the canine was visible in her mouth (Figures 7 and 8), so the fishing rod was replaced with a vestibular rigid arch, welded on the band, to continue the orthodontic traction. Pre-informed brackets and straight archwires were used; for the first 15 days a 0.356mm (0.014 inch) nickel-titanium alloy (NiTi) archwire was used; then it was replaced with a 0.016×0.022 inch NiTi archwire (Figure 8). When the canine was present in the oral cavity, a bracket was added to it and linked directly to the arch by an elastic ligation. To keep the space in the arch for the canine, since it was not aligned, we used an open coil spring (Figure 9). Finally after approximately 18 months, the canine was well positioned in the arch (Figure 10). At this time, she was advised that she needed an attached gingiva graft on her restored tooth to improve esthetics and the periodontal health compromised by the treatment (Figure 11).

**Figure 12:**
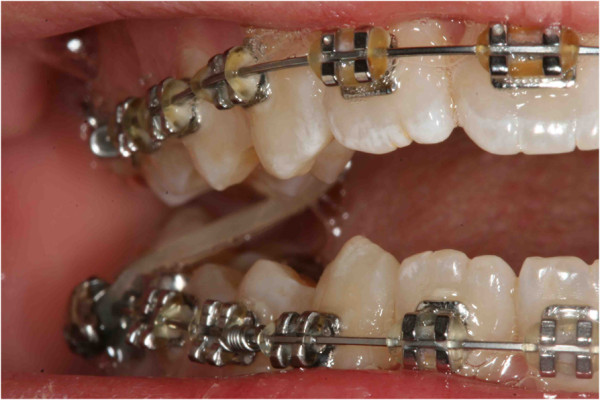
Criss-cross elastic to improve the intercuspidation between the upper right first molar and the lower right first molar.

**Figure 13:**
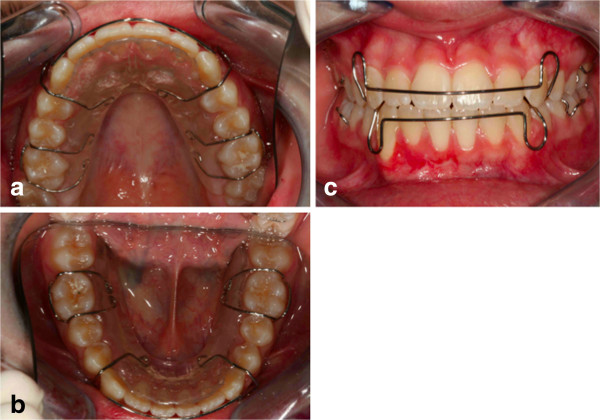
**Retention was established with removable appliances on the upper arch (a) and the lower arch (b), to maintain the obtained result (c).** After orthodontic treatment finished and the canine positioned in an acceptable way in the dental arch, the patient was advised that she needed an attached gingiva graft on the restored tooth to improve esthetics and the periodontal health compromised by the treatment.

**Figure 14:**
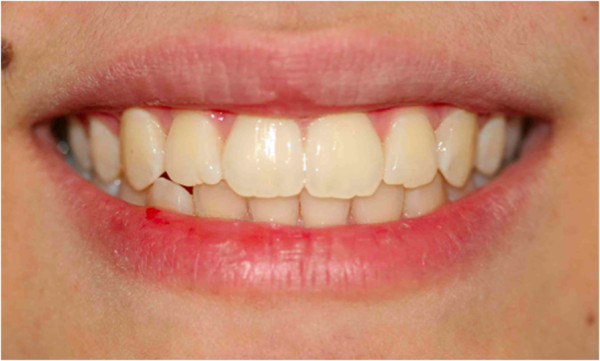
Post-treatment photograph of the smile.

**Figure 15:**
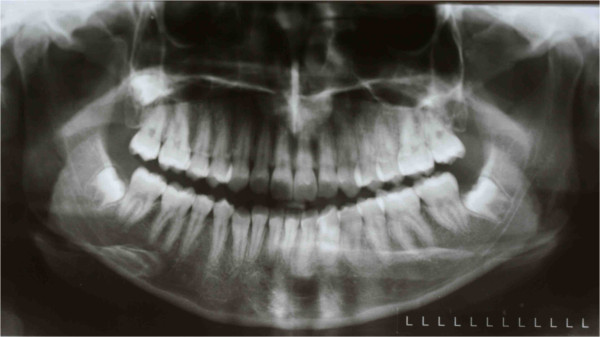
Post-treatment orthopantomograph.

**Figure 16:**
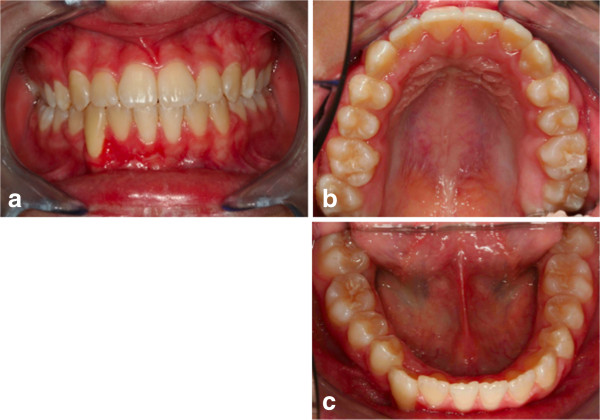
**Post-treatment intraoral photographs: (a) frontal view; (b) upper occlusal view; (c) lower occlusal view.** One year after, only a partial recurrence was observed in the position of the upper first right molar, as the patient had not observed the restraint protocol. The periodontal surgery (attached gengiva graft of the lower right canine) has not been performed as requested by the same patient. The need for an attached gingiva graft on the restored tooth remained.

The last step to improve the intercuspidation was the use of criss-cross elastics between her upper right first molar and her lower right first molar (Figure 12). When an acceptable occlusion with adequate root angulation had been achieved, the fixed appliance was removed.Retention was established with removable appliances (Figure 13). Then, here is the smile of the girl (Figure 14).The post-treatment radiographic view (Figure 15) showed that the roots of her teeth in her upper arch were well angulated and aligned. No apical root resorption was evident on the radiograph. The midline as well as the overjet and overbite had been maintained during the treatment. Periodontal health was not compromised. One year after treatment follow-up there was no obvious relapse. Her midline, overjet, and overbite are still in good position. No tooth morbidity is evident. One year after debonding, only a partial recurrence was observed in the position of the upper first right molar, as she had not observed the restraint protocol (Figure 16). Also the periodontal problem (the lack of attached gengiva) at the level of the lower right canine was confirmed. Her gums are healthy, and although the lack of attached gengiva in the canine region is intact, she is satisfied with the treatment results.

### Clinical case 2

**Figure 17:**
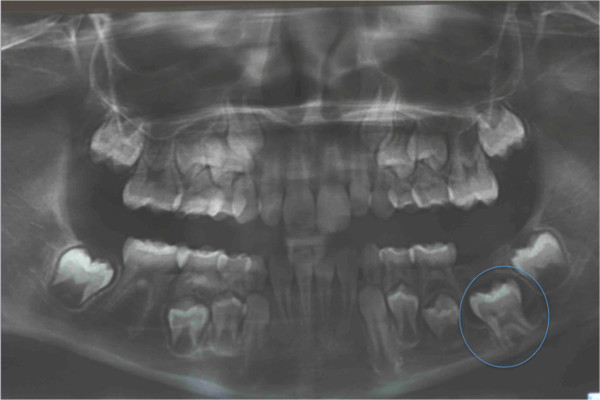
First orthopantomograph, before the patient came to our attention: a general dentist had suggested the extraction of the impacted left lower first molar (in the blue circle), after viewing this orthopantomograph.

A 9-year-old Italian girl was referred by her pediatrician because her lower first permanent left molar was not present in her dental arch. Before coming to our attention a general dentist had suggested extraction of the element, after viewing the orthopantomograph (Figure 17) in which the apical third of the roots showed a marked angulation with respect to the long axis of the root itself (Figure 17). However, her pediatrician, consulted for a medical visit by her mother, hearing the proposal to extract a permanent tooth, advised her to consult an orthodontist, a dentist who specializes in orthodontics before the surgical extraction.

She was in good health, and her dental and medical history was unremarkable with only the usual childhood maladies.

**Figure 18:**
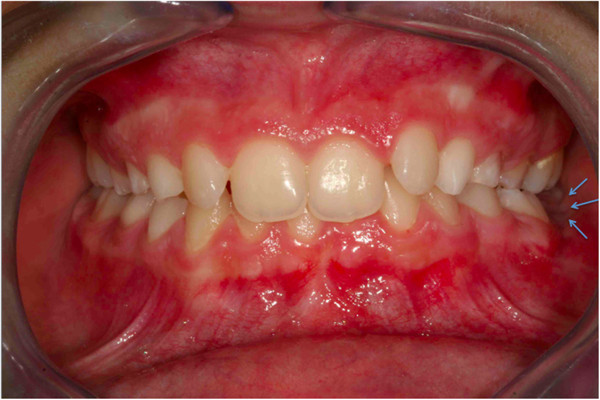
**Pretreatment intraoral frontal photograph.** In correspondence with the missing tooth you see an “empty area” in the occlusion of the patient (indicated by the blue arrows).

**Figure 19:**
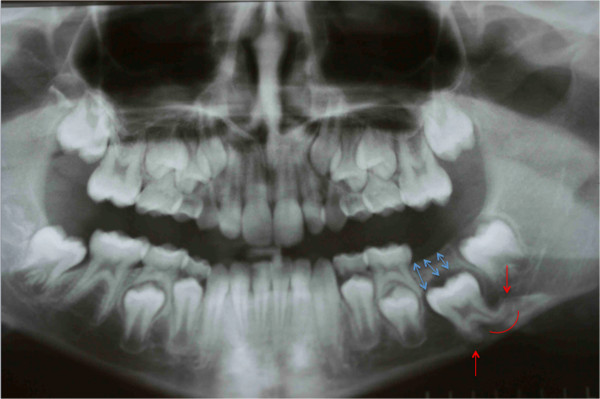
Second orthopantomograph prescribed to the patient by us before our intervention to assess the evolution of the clinical case and formulate a new plan of treatment: the situation was even worse; the roots were all sizes and showed closed apexes (red arrows), and the presence of bone above was increased with respect to the first evaluation (blue arrows).

An extraoral clinical examination disclosed a symmetrical face with balanced vertical thirds.She has a dental-skeletal class I with mandibular normal divergence, with no bad habits; her cephalometric values are all normal, even her lower and upper incisors are normal-inclined. Her profile was standard for Italian people. An intraoral examination revealed that her dental midlines were not concordant with each other and with her face and no mandibular shift was detected on closure. Except for some lower incisor crowding and deep-bite, the overall occlusion was fair with acceptable overjet (Figure 18). The molar relationship was Class I at right and left sides. We performed an orthopantomogram (OPT) 1 year after her first visit to the other dentist and we noticed that the situation was even worse; the roots were all sizes and showed closed apexes, and the presence of bone above was increased with respect to the first evaluation (Figure 19). After careful evaluation of this case, we decided to treat this impacted tooth orthodontically. Full mouth orthodontic treatment was suggested. Before the beginning of the therapy, the patient and her parents were informed about the difficulties of the treatment and the recovery of tooth. It was our goal to treat this case with a non-extraction orthodontic approach using upper and lower orthodontic appliances, while doing our best to correct the impacted tooth, maintaining the profile and reaching as good a final occlusion as possible.

The objectives of orthodontic treatment for this patient were to bring the impacted mandibular left first molar into her dental arch, level and align her arches, maintain her normal overjet, improve her overbite, and achieve a bilateral Class I canine and molar occlusion.

**Figure 20:**
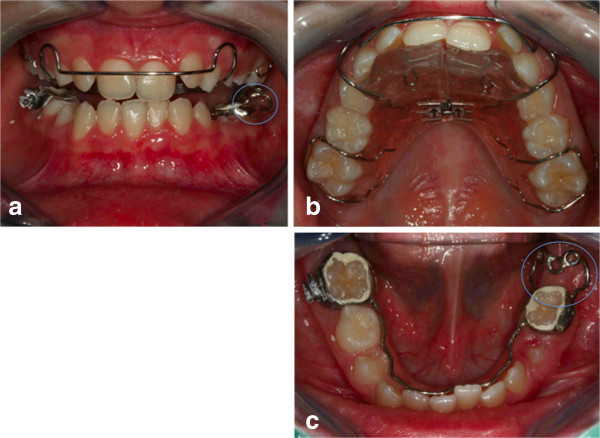
**The appliances used. (a)** Intraoral frontal photographs. **(b)** Lingual arch. **(c)** Upper appliance. The lingual arch shows an extension distal to the deciduous molar, to allow the extrusion of the retained first molar (blue circles).

**Figure 21:**
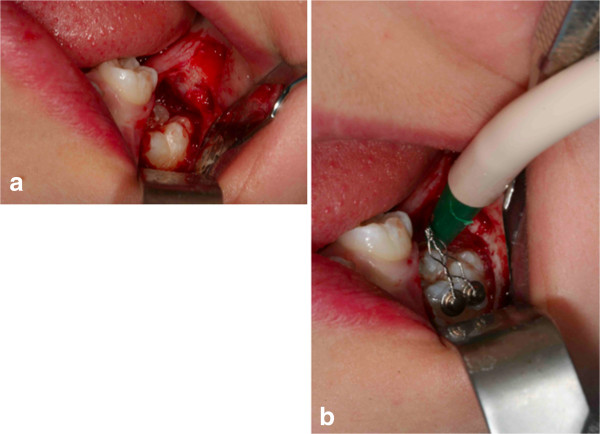
**Surgery (a) and placement of buttons (b).** Two buttons were bonded on the molar crown in order to prevent the reopening of the surgical site in case one of the buttons came off accidentally during orthodontic treatment.

**Figure 22:**
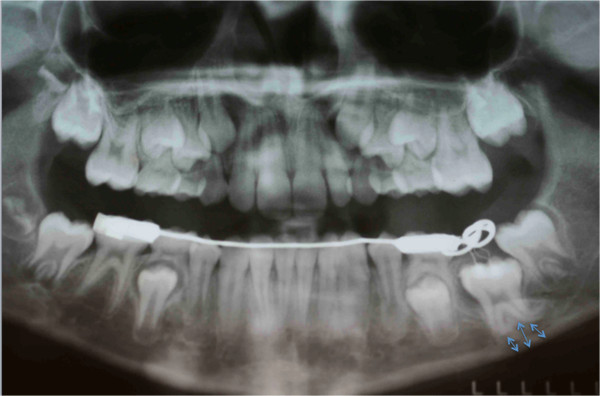
Orthopantomograph 3 months after surgery: it confirmed that the tooth was moving, as evidenced by the increase in the distance between the lower edge of the jaw and the roots of the impacted tooth (blue arrows).

**Figure 23:**
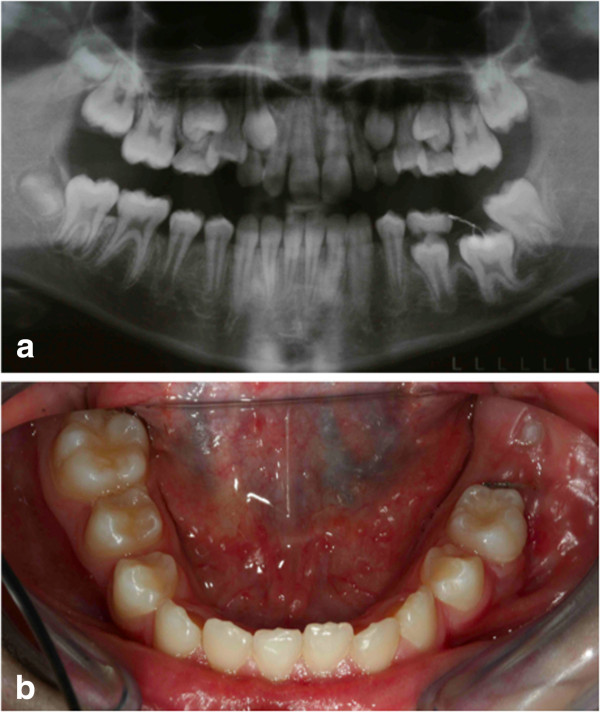
**Ten months after surgery; when the second molar was erupting, and the first molar was impacting against it (as seen in the orthopenthomograph, a), it was decided to wait for the eruption of the second molar (b) before continuing the treatment.** So the lingual arch was eliminated to avoid the mesialization of the second molar.

**Figure 24:**
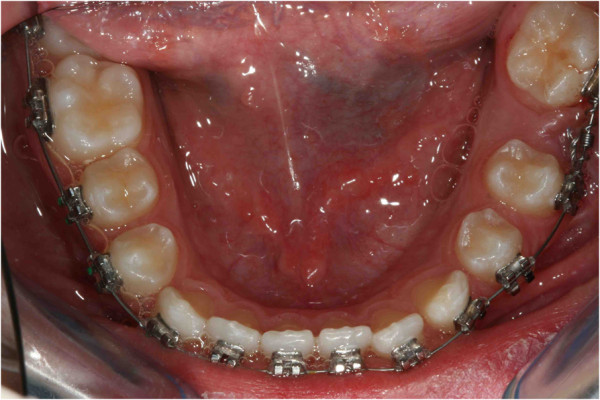
One year later.

**Figure 25:**
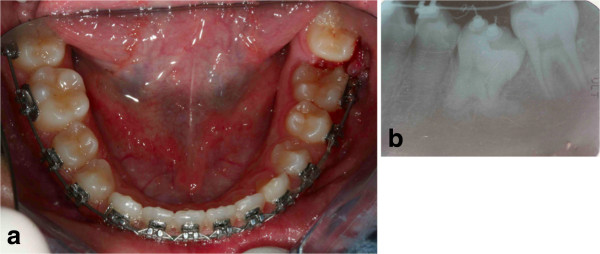
**After 6 months. (a)** Intraoral photo. **(b)** Intraoral radiograph. Pre-informed brackets and straight archwires were used; approximately 6 months after the beginning of the fixed treatment, the tooth appeared in the dental arch.

**Figure 26:**
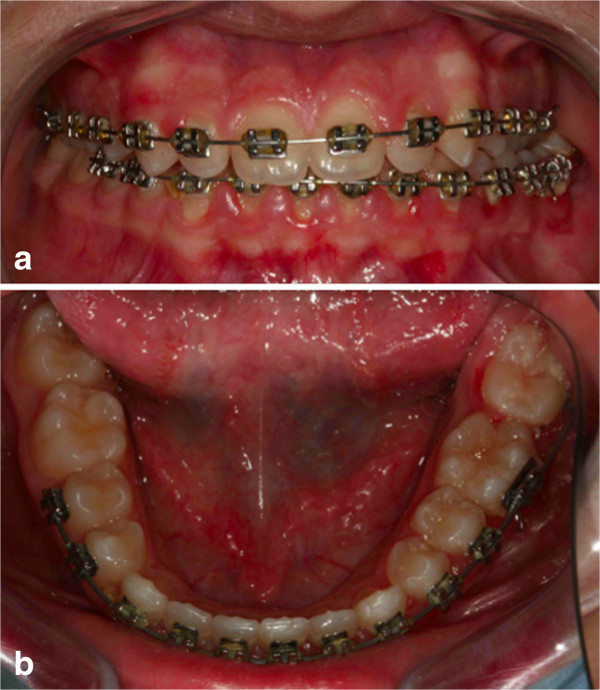
**The molar is extruded. (a)** Frontal intraoral. **(b)** Occlusal photo.

**Figure 27:**
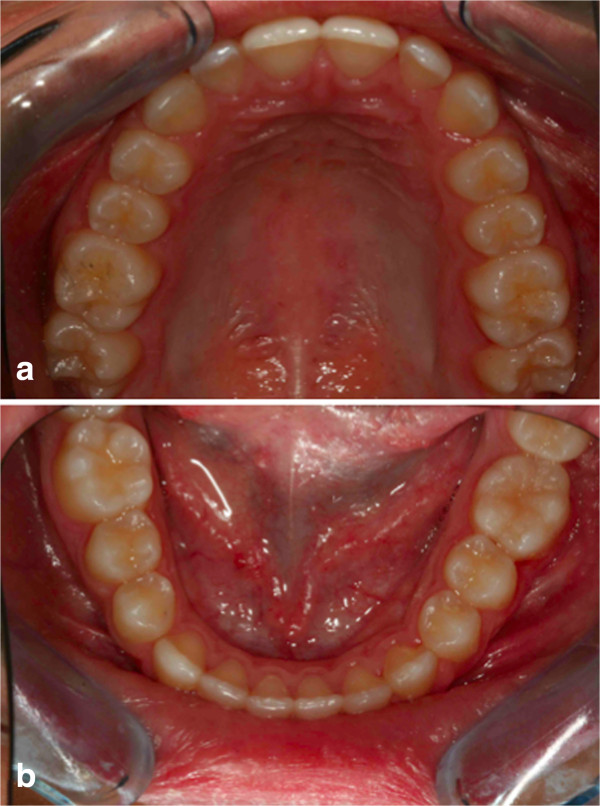
Post-treatment occlusal photographs; (a) upper occlusal view; (b) lower occlusal view.

**Figure 28:**
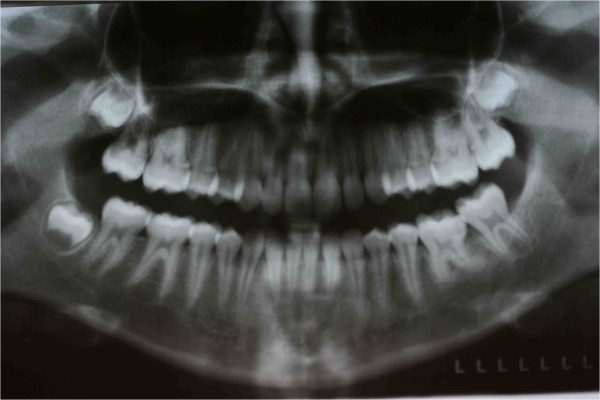
Post-treatment radiograph.

**Figure 29:**
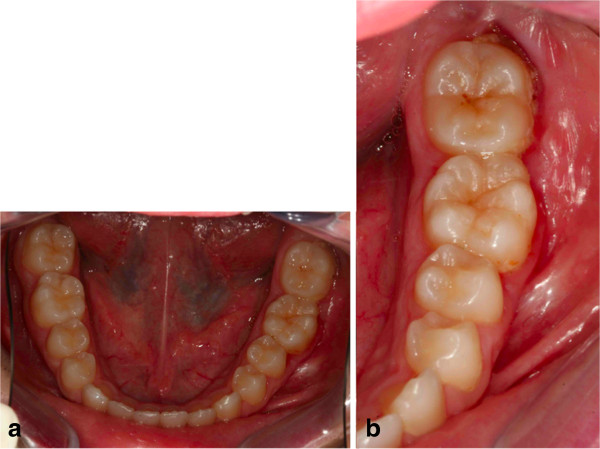
**Intraoral photographs 1 year post-treatment. (a)** lower occlusal view; **(b)** the treated teeth; no tooth morbidity is evident. The gums are healthy, and the gingival attachment in the molar region is intact.

As the first step, a lingual arch was designed and cemented on her lower left deciduous molar and lower right first molar; the lingual arch shows an extension distal to the deciduous molar, to allow the extrusion of the retained first molar. The upper arch was prepared using a Schwarz plate with expansion to improve the dental arch contraction (Figure 20). Before the bonding of the arch, surgical intervention was performed to place the orthodontic bracket on the impacted molar crown (Figure 21a). After the opening session, two buttons were bonded on the molar crown (Figure 21b). Three months after surgery, an OPT was taken as a control and this confirmed that the tooth was moving, as evidenced by the increase in the distance between the lower edge of her jaw and the roots of the impacted tooth (Figure 22). Then, as the second molar was erupting, and the first molar was impacting against it, it was decided to wait for the eruption of the second molar before continuing the treatment. So the lingual arch was eliminated to avoid the mesialization of the second molar (Figure 23). When the lingual arch was removed, the functional therapy in the upper arch was continued. After a year, we bonded the lower dental arch. We then positioned an open coil spring to increase the space for the molar. The molar was then ligated directly to the archwire (Figure 24). Then, the molar was tied directly to the archwire with constant and light force. We used pre-informed brackets and straight archwires. In the first session we used a NiTi (0.014 inch) archwire; we then replaced it with a (0.016×0.022 inch) NiTi archwire. Approximately 6 months after treatment the tooth appeared in the dental arch (Figure 25), and some weeks later perfectly extruded (Figure 26). Criss-cross elastics were used to improve the intercuspidation. After approximately a year, the appliance was removed (Figure 27). The total treatment time for this patient was 24 months. Retention was established with a removable plate. The post-treatment panoramic view showed that the roots of her teeth were well angulated and aligned. No apical root resorption was evident on a radiograph (Figure 28). The orthodontic treatment allowed the correct placement of midline, overjet and overbite. The radicular anomalies of the impacted first molar could be due to the impact of the tooth near the lower contour of her mandible. Perhaps, if the treatment was made a year before, this anomaly could be less evident. The periodontium of the tooth during treatment showed some alterations of an inflammatory nature, which were treated with sessions of hygiene and good oral home health. One year post-treatment follow-up there was no obvious relapse (Figure 29). No tooth morbidity is evident. Her gums are healthy, and the gingival attachment in her molar region is intact. She is satisfied with the treatment results [26].

## Discussion

### Clinical case 1

An alternative treatment for this young girl was the extraction of the impacted tooth and rehabilitation with a prosthesis, such as a bridge, removable denture, or implants [5,6].

The advantages of treating without extraction were both functional and esthetic, because in a young patient it is not possible to achieve an implant solution [4]. A normal complement of anterior teeth would be more attractive and would be most likely to achieve functional ideals (elimination of nonworking contacts, and achievement of ideal overjet and overbite) [4].

The disadvantages of the orthodontic treatment included prolonged treatment time and the possibility of failure. Fortunately, these problems did not occur in this patient.

In the absence of any therapy, the impacted teeth could have become ankylosed, with lost vitality, or succumbed to root resorption, or all of these [3,4].

From a biomechanical point of view, if sufficient space for the canine exists or has been created in the dental arch, it is desirable to deliver a light, point force in the occlusal direction [4,12]. The inclusion of many teeth in the orthodontic device also helps to distribute the unwanted intrusive side effects among a larger cumulative root surface area and thus minimize localized deleterious effects (the concept of orthodontic anchorage) [4,13].

Also, application of a more rigid and larger main archwire, plus an open coil spring, helps to hold the canine space and to prevent intrusion of the adjacent teeth during canine extrusion [4].

### Clinical case 2

Correction of impaction of the lower first molar has not been adequately presented in the literature. This particular disturbance is rather difficult to prevent because of its multifactorial and often hypothetical etiology [3], yet a careful orthodontic treatment is required according to the *primum non nocere* (first, do no harm) principle. An alternative treatment for this young female girl included the extraction of the impacted tooth and the rehabilitation with an implant [5,6]. The advantage of the orthodontic treatment was functional because in a young patient an implant solution is not possible.

The disadvantages of the orthodontic treatment included prolonged treatment time and the possibility of failure [3,4,13]. The impacted teeth could have become ankylosed, lost vitality, or succumbed to root resorption [3,4,13]; fortunately, these problems did not occur in this patient.

The therapeutic goal obtained with these two patients is probably linked to their young ages of 13 years and 9 years [4].

In general, these cases seem to suggest that the orthodontic treatment of impacted teeth with difficult practicability can be justified in very young adolescents (13- to 14-years old) or children (9-years old): in these cases the treatment duration seems to be acceptable and the results good. Early diagnosis has a strategic importance in these cases [3,4]. A pediatrician’s early suspicion of impacted teeth can be strategic; dentists can then complete diagnosis and prognosis with an adequate and successful treatment. Often the parents of a young patient can ask their pediatrician to assess which is the right time to refer the child for a dental visit, or even orthodontics. This is the reason why it is better that the pediatrician is aware of the complications that arise from the inclusion of permanent teeth, which can be prevented and cured when the patient is a child. In the most difficult clinical cases of impacted teeth it is very important to have an early diagnosis, which is essential to plan the treatment and achieve success.

## Conclusions

Even complex impacted teeth can be retrieved without causing damage to the other teeth already in the dental arch. Considering individual cases, evaluating the particular circumstances and planning suitable treatment for each individual situation is the key to success.

The pediatrician is in a strategic position to give an early diagnosis through a child’s medical history and by counting the child’s teeth.

## Consent

Written informed consent was obtained from the patients’ legal guardians for publication of this case report and accompanying images. A copy of the written consents is available for review by the Editor-in-Chief of this journal.

## Competing interests

The authors declare that they have no competing interests.

## Authors’ contributions

ST organized the data, made the analysis of results, the discussion of results, the figures and drafted the manuscript; MTD organized the data, made the analysis of results, the discussion of results, the figures and drafted the manuscript; EM coordinated the recording of data and the review of the literature; GG reviewed the literature and helped in the treatment of the patients; SM helped in the review of the literature; ML organized the data, made the analysis of results, the discussion of results, the figures and drafted the manuscript; VC and GM treated the patients and coordinated the drafting of the manuscript. All the authors read and approved the final manuscript.

## Authors’ information

ST is Researcher at the University Vita-Salute San Raffaele, Milano, Italy; EM, SM and GG are Researchers at the Department of Life, Health and Environmental Sciences, University of L’Aquila, Italy; MTD, and ML are PhD students at the University of L’Aquila, Italy; VC is Associate Professor at the University Tor Vergata, Rome, Italy; GM is Full Professor at the University of L’Aquila, Italy and is the Head of the School in Orthodontics at the same university.
